# Differences between primary peritoneal serous carcinoma and advanced serous ovarian carcinoma: a study based on the SEER database

**DOI:** 10.1186/s13048-021-00788-y

**Published:** 2021-02-27

**Authors:** Xiaoduo Li, Qiao Yang, Mingjing Chen, Changqing Yang, Jianfen Gu, Qiang Dong, Guangrong Yang

**Affiliations:** 1 Department of Obstetrics and Gynecology, Qijiang Maternal and Child Health Hospital, Qijiang, Chongqing, China; 2 Department of Ultrasound, The 941st Hospital of the PLA Joint Logistic Support Force, Xining, Qinghai China; 3 Department of Infectious Diseases, The First Affiliated Hospital of Chongqing Medical University, Chongqing, Chongqing, China; 4 Department of Oncology, Qijiang Hospital of the First Affiliated Hospital of Chongqing Medical University, Qijiang, Chongqing, 401420 China; 5 Department of Nutrition, Qijiang Hospital of the First Affiliated Hospital of Chongqing Medical University, Qijiang, Chongqing, China; 6 Department of General Medicine, Qijiang Hospital of the First Affiliated Hospital of Chongqing Medical University, Qijiang, Chongqing, 401420 China

**Keywords:** Primary peritoneal serous carcinoma, Advanced serous ovarian carcinoma, Clinical features, Overall survival, SEER

## Abstract

**Objective:**

This study aimed to compare clinical features and overall survival (OS) between patients with primary peritoneal serous carcinoma (PPSC) and those with advanced serous ovarian carcinoma (ASOC) and to identify prognostic factors.

**Methods:**

Patients diagnosed with PPSC and ASOC from 2010 to 2015 from the Surveillance, Epidemiology, and End Results (SEER) database were enrolled. Pearson’s chi-square test was used to compare clinical features. The primary endpoint was OS. The Kaplan–Meier method and log–rank test were used to perform the survival analysis. Propensity score matching was also conducted. Univariate, multivariate and subgroup analyses were performed using the Cox proportional hazards model.

**Results:**

A total of 708 PPSC patients and 7610 ASOC patients were enrolled. The clinical features of PPSC patients were noticeably different from those of ASOC patients. The survival analysis showed that PPSC patients had poorer outcomes than ASOC patients. Even after the clinical features were balanced, PPSC patients still had poorer survival. Univariate and multivariate analyses indicated that older age, higher tumor grade and advanced American Joint Committee on Cancer stage were adverse prognostic factors in both groups, while surgery and chemotherapy were protective factors. A subgroup analysis demonstrated that most factors favored ASOC patients. The total distant metastasis rates of PPSC and ASOC were similar. Liver or lung metastasis was common, but bone and brain metastases were rare. A higher proportion of liver metastasis was observed in the ASOC group.

**Conclusion:**

The clinical features and survival outcomes between PPSC patients and ASOC patients are clearly different, and PPSC is more aggressive than ASOC.

**Supplementary Information:**

The online version contains supplementary material available at 10.1186/s13048-021-00788-y.

## Introduction

Primary peritoneal carcinoma (PPC) originates in the peritoneum and leads to diffuse cancerous changes in the abdominal and pelvic cavities [1, 2]. Clinically, female patients with PPC resemble patients with advanced epithelial ovarian cancer (EOC). However, the ovaries of PPC patients are rarely affected, and when they are, only the surfaces are affected [1, 3]. The incidence of PPC is low. It was reported that the incidence of EOC is 4 times more than that of PPC [4].

According to the World Health Organization (WHO), the pathological types of PPC and EOC include serous carcinoma (SC), mucinous carcinoma (MC), endometrioid carcinoma (EC), clear-cell carcinoma (CCC), transitional-cell Brenner tumor, mixed, and undifferentiated type [5, 6]. SC is divided into two subtypes: high-grade serous carcinoma (HGSC) and low-grade serous carcinoma (LGSC). HGSC accounts for 85–90% of all SCs, and most HGSCs have p53 mutations [6, 7]. The prognosis of HGSC is poor because most HGSC patients are at an advanced stage of disease at diagnosis. Histological, molecular and genetic evidence has shown that approximately 40–60% of HGSCs of the ovary or peritoneum originate from the fimbrial end of the fallopian tube [8]. LGSC is a kind of slow-growing tumor with a good prognosis. Most LGSCs have KRAS and/or BRAF mutations [9, 10].

According to the National Comprehensive Cancer Network (NCCN)-Clinical Practice Guidelines in Oncology (NCCN Guidelines), the therapeutic principle of PPC is similar to that of EOC, with surgery and chemotherapy as the main treatment regimen [11]. One study investigated the epidemiological differences between PPC and EOC [12]. The results showed that PPC patients were older and experienced later menarche. No other significant differences were found. Halperin reported a worse three-year survival in PPC patients than in EOC patients [8]. Another study demonstrated that patients with LGSC of the peritoneum had a significantly better progression-free survival (PFS) and overall survival (OS) than patients with LGSC of the ovary [13]. In the study by Gurkan, no significant differences were found in the disease-free survival and OS among patients with ovarian cancer, PPC and tubal cancer [14].

As serous carcinoma is the most common subtype of both PPC and EOC, some studies have focused on comparisons between primary peritoneal serous carcinoma (PPSC) and serous ovarian carcinoma (SOC). One such study enrolled 22 patients with the papillary subtype of PPSC and 63 stage III/IV patients with the papillary subtype of SOC. The results demonstrated that the clinical characteristics between the two groups were similar. Additionally, the median disease-free interval, OS, and 5-year survival rates were similar between the two groups [15]. Another study also found no obvious differences in survival or response rate to chemotherapy and surgery [16]. Due to the low incidence of PPC, most studies have limited sample sizes. The United States National Cancer Institute (NCI) established the Surveillance, Epidemiology and End Results (SEER) database in 1973. SEER is one of the world’s recognized authoritative sources of cancer patient follow-up data, as it provides reliable data support for clinical research. Thus, in this study, we aimed to analyze differences between PPSC and advanced SOC (ASOC) in a large sample, including clinical features, OS and distant metastasis and to identify prognostic factors.

## Materials and methods

### Patient selection and clinical features

We used the latest submission of the SEER database, which was released in April 2019 and includes all reportable cancer cases from 18 population-based cancer registries (1975–2016) [17]. The software used was SEER*Stat (version 8.3.6). Patients were determined to be eligible and were included according to the following criteria: PPSC and ASOC cases diagnosed between 2010 and 2015, primary tumor located in the ovary or peritoneum, and only one primary tumor. Cases diagnosed by death certificate or autopsy were not included. All cases were identified by histology codes according to the International Classification of Diseases for Oncology, 3rd Edition (ICD-O-3). The histology codes used in this study were as follows: 8441/3, 8460/3, 8462/3, 8461/3, and 8463/3. The clinical features assessed in this study included age, race, marital status, tumor grade, American Joint Committee on Cancer (AJCC) stage, chemotherapy, surgery, overall survival, and status of life records. Patients with unknown AJCC stage and tumor grade were excluded. In addition, as all PPSC patients were AJCC stage III and IV, SOC patients with AJCC stage I and II were excluded.

### Outcome measurement

The primary endpoint was OS, which was defined as the time interval from the diagnosis of cancer to death from any cause or the last follow-up. Patients who were still alive at the last follow-up were considered censored cases. The final follow-up date was 31 December 2016.

### Statistical analysis

In this study, Pearson’s chi-square test was used to compare the clinical feature differences between PPSC and ASOC. Survival curves were plotted using the Kaplan–Meier method, and survival differences were determined using the log-rank test. Univariate and multivariate analyses were performed, and adjusted hazard ratios (HRs) with 95% confidence intervals (CIs) were calculated using the Cox proportional hazards model. For further analysis, we matched each PPSC case with two ASOC cases by the propensity score matching (PSM) method, and all clinical features were considered. To perform the subgroup analysis, an unadjusted Cox proportional hazard model was used to calculate HRs with 95% CIs of PPSC versus matched ASOC, and a forest plot was used to better illustrate the effect of each prognostic factor on OS.

A two-sided *P* value < 0.05 was considered statistically significant. PSM was performed in R studio (version 3.5.2) with the MatchIt package. All statistical analyses were performed using SPSS version 23.0 (IBM, Armonk, NY, USA).

## Results

### Comparison of the baseline clinical features between PPSC and ASOC

In all, 8318 patients, including 708 PPSC patients and 7610 ASOC patients, were enrolled. Obvious differences were found in the baseline clinical features between PPSC and ASOC (Table 1). The age distribution was completely different between PPSC and ASOC patients. Compared with the ASOC group, the PPSC group had a significantly lower percentage of patients aged ≤49 years (5.8% vs. 19.0%) but had a higher percentage of patients aged ≥70 years (39.5% vs. 27.6%, *P* <  0.001). Additionally, in terms of race, the PPSC group had a lower percentage of black patients (4.4% vs. 7.4%, *P* = 0.003). In addition, the distribution of tumor grade was different between PPSC and ASOC. Moreover, patients who underwent surgery accounted for 91.7% in the PPSC group and 94.8% in the ASOC group. No significant differences were found in marital status, AJCC stage or chemotherapy.

**Table 1:** Baseline clinical features difference between PPSC and ASOC patients

Clinical features	PPSC *N* = 708(%)	ASOC *N* = 7610(%)	Total *N* = 8318(%)	*P* value
Age				< 0.001
≤ 49	41 (5.8)	1447 (19.0)	1488 (17.9)	
50–69	387 (54.7)	4363 (57.3)	4750 (57.1)	
≥ 70	280 (39.5)	2100 (27.6)	2380 (28.6)	
Race				0.003
White	600 (84.7)	6393 (84.0)	8365 (84.1)	
Black	31 (4.4)	560 (7.4)	693 (7.0)	
Others	77 (10.9)	657 (8.6)	886 (8.9)	
Marital status				0.740
Married	398 (56.2)	4164 (54.7)	4562 (54.8)	
Not married	283 (40.0)	3153 (41.4)	3436 (41.3)	
Unknown	27 (3.8)	293 (3.9)	391 (3.8)	
Tumor grade				0.003
I	18 (2.5)	179 (2.4)	197 (2.4)	
II	88 (12.4)	634 (8.3)	722 (8.7)	
III	318 (44.9)	3539 (46.5)	3857 (46.4)	
IV	284 (40.1)	3258 (42.8)	3542 (42.6)	
AJCC stage				0.105
III	466 (65.8)	5234 (68.8)	5700 (68.5)	
IV	242 (34.2)	2376 (31.2)	2618 (31.5)	
Chemotherapy				0.605
Yes	626 (88.4)	6678 (87.8)	7304 (87.8)	
No/Unknown	82 (11.6)	932 (12.2)	1014 (12.2)	
Surgery				< 0.001
Yes	649 (91.7)	7215 (94.8)	7864 (94.5)	
No/Unknown	59 (8.3)	395 (5.2)	454 (5.5)	

*PPSC* primary peritoneal serous carcinoma, *ASOC* advanced serous ovarian cancer, *AJCC* American Joint Committee on Cancer

### Survival analysis

**Fig. 1:**
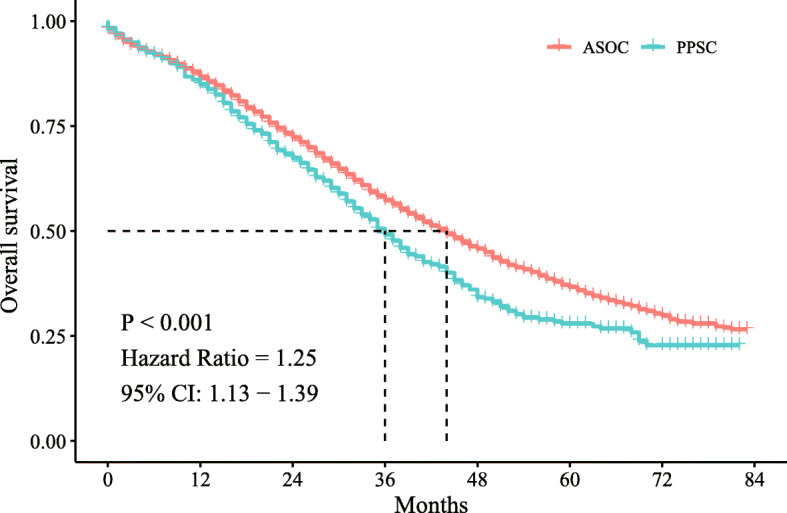
Kaplan–Meier curves for overall survival. The dotted lines indicate the median overall survival of patients with PPSC and ASOC. PPSC, primary peritoneal serous carcinoma; ASOC, advanced serous ovarian cancer; CI, confidence interval

A poorer OS was observed in the PPSC group than in the ASOC group (*P* <  0.001, Fig. 1). The median OS was 36.0 months (95% CI, 33.3–38.7) in the PPSC group and 44.0 months (95% CI, 42.6–45.4) in the ASOC group. The HR for death was 1.25 (PPSC vs. ASOC, 95% CI, 1.13–1.39; *P* <  0.001).

### Univariate and multivariate analyses

**Fig. 2:**
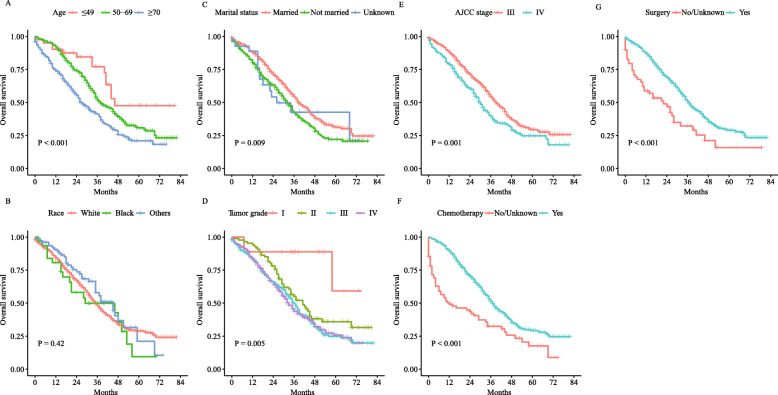
Kaplan–Meier curves for the univariate analysis of PPSC: **a**, Age; **b**, Race; **c**, Marital status; **d**, Tumor grade; **e**, AJCC stage; **f**, Chemotherapy; **g**, Surgery. PPSC, primary peritoneal serous carcinoma; AJCC, American Joint Committee on Cancer

**Fig. 3:**
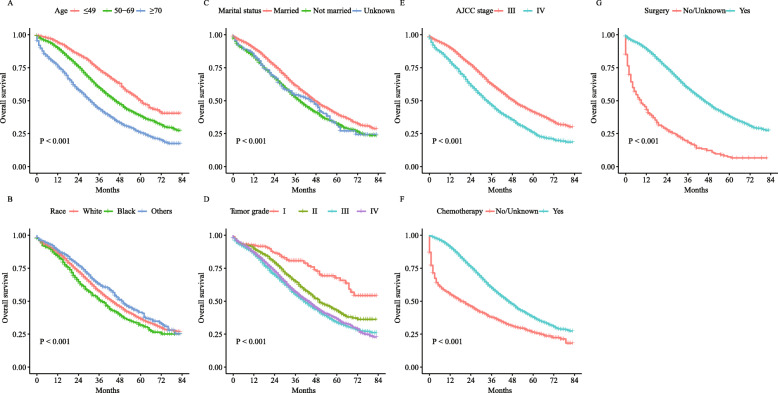
Kaplan–Meier curves for the univariate analysis of ASOC: **a**, Age; **b**, Race; **c**, Marital status; **d**, Tumor grade; **e**, AJCC stage; **f**, Chemotherapy; **g**, Surgery. ASOC, advanced serous ovarian cancer; AJCC, American Joint Committee on Cancer

Univariate and multivariate analyses were performed to investigate the impact of certain clinical features on the OS of patients with PPSC or ASOC. For both PPSC and ASOC, older age, higher tumor grade and advanced AJCC stage were adverse prognostic factors in both the univariate and multivariate analyses, while surgery and chemotherapy were protective factors (Tables 2 & 3). For PPSC, unmarried status was an adverse prognostic factor compared with married status in the univariate analysis, but this finding did not reach statistical significance in the multivariate analysis. No difference was found among patients of different races (Table 2). For ASOC, in terms of marital status, unmarried status was an adverse prognostic factor in both the univariate and multivariate analyses. Additionally, black race was an adverse prognostic factor compared with white race in both the univariate and multivariate analyses (Table 3). In addition, survival curves of the univariate analysis of PPSC and ASOC were plotted (Figs. 2 & 3).

**Table 2:** Risk factors for overall survival of PPSC patients by univariate and multivariate analyses

Clinical features	Univariate analysis			Multivariate analysis		
	HR	95% CI	*P* value	HR	95% CI	*P* value
Age						
≤ 49	Reference	–	–	Reference	–	–
50–69	1.70	0.99–2.92	0.056	1.66	0.96–2.85	0.069
≥ 70	2.71	1.57–4.67	< 0.001	2.49	1.44–4.31	0.001
Race						
White	Reference	–	–			
Black	1.20	0.75–1.90	0.445			
others	0.84	0.59–1.18	0.317			
Marital status						
Married	Reference	–	–	Reference	–	–
Not married	1.37	1.12–1.68	0.003	1.22	0.99–1.51	0.061
Unknown	1.31	0.76–2.25	0.330	1.13	0.65–1.96	0.676
Tumor grade						
I	Reference	–	–	Reference	–	–
II	3.65	1.15–11.82	0.028	5.20	1.61–16.87	0.006
III	4.89	1.56–15.32	0.006	7.61	2.38–24.28	0.001
IV	5.00	1.59–15.68	0.006	7.94	2.48–25.38	< 0.001
AJCC stage						
III	Reference	–	–	Reference	–	–
IV	1.41	1.14–1.73	0.001	1.36	1.11–1.68	0.004
Chemotherapy						
No/Unknown	Reference	–		Reference	–	–
Yes	0.48	0.36–0.64	< 0.001	0.36	0.27–0.48	< 0.001
Surgery						
No/Unknown	Reference	–	–	Reference	–	–
Yes	0.50	0.36–0.70	< 0.001	0.61	0.44–0.86	0.005

*PPSC* primary peritoneal serous carcinoma, *HR* hazard ratio, *CI* confidence interval, *AJCC* American Joint Committee on Cancer

**Table 3:** Risk factors for overall survival of ASOC patients by univariate and multivariate analyses

Clinical features	Univariate analysis			Multivariate analysis		
	HR	95% CI	*P* value	HR	95% CI	*P* value
Age						
≤ 49	Reference	–	–	Reference	–	–
50–69	1.50	1.35–1.67	< 0.001	1.38	1.24–1.54	< 0.001
≥ 70	2.45	2.19–2.74	< 0.001	1.97	1.75–2.21	< 0.001
Race						
White	Reference	–	–	Reference	–	–
Black	1.20	1.07–1.36	0.002	1.19	1.05–1.34	0.005
others	0.87	0.76–0.98	0.022	0.90	0.79–1.02	0.083
Marital status						
Married	Reference	–	–	Reference	–	–
Not married	1.32	1.24–1.41	< 0.001	1.21	1.13–1.29	< 0.001
Unknown	1.28	1.08–1.52	0.004	1.12	0.94–1.32	0.205
Tumor grade						
I	Reference	–	–	Reference	–	–
II	1.87	1.38–2.53	< 0.001	1.86	1.38–2.53	< 0.001
III	2.47	1.86–3.29	< 0.001	2.29	1.72–3.05	< 0.001
IV	2.34	1.76–3.11	< 0.001	2.35	1.77–3.13	< 0.001
AJCC stage						
III	Reference	–	–	Reference	–	–
IV	1.63	1.53–1.74	0.001	1.47	1.37–1.57	< 0.001
Chemotherapy						
No/Unknown	Reference	–		Reference	–	–
Yes	0.48	0.44–0.52	< 0.001	0.50	0.46–0.55	< 0.001
Surgery						
No/Unknown	Reference	–	–	Reference	–	–
Yes	0.24	0.21–0.27	< 0.001	0.33	0.29–0.37	< 0.001

*ASOC* advanced serous ovarian cancer, *HR* hazard ratio, *CI* confidence interval, *AJCC* American Joint Committee on Cancer

### Subgroup analysis

A 1:2 (PPSC: ASOC) matched case-control analysis was performed to exclude the effect of clinical feature biases on the survival analysis. A total of 708 PPSC patients and 1416 matched ASOC patients were included. No difference in clinical features was observed between the matched groups (Supplementary Table 1). The Kaplan–Meier survival analysis showed that the PPSC group had a poorer OS than the matched ASOC group (*P* = 0.004). The median OS was 36.0 months (95% CI 33.3–38.7) in the PPSC group and 43.0 months (95% CI, 39.7–46.3) in the matched ASOC group (HR 1.20, 95% CI 1.06–1.36; *P =* 0.004) (Supplementary Fig. 1).

**Fig. 4:**
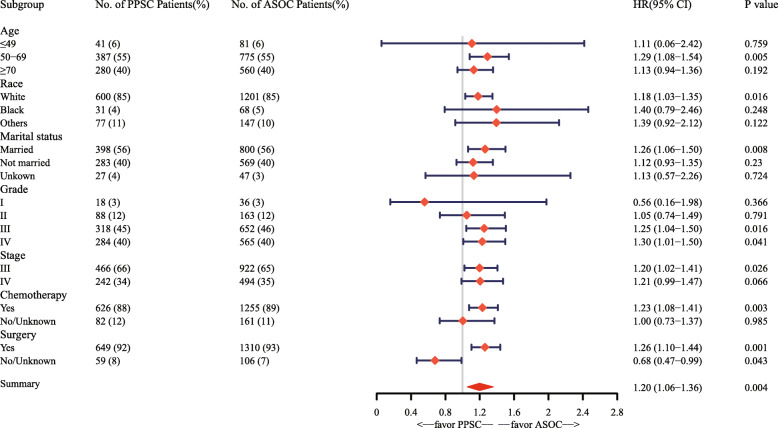
Forest plot of the HR for PPSC versus matched ASOC in the subgroup analysis. The circle and line segments represent the HR and 95% CI of each subgroup. A HR > 1.00 indicates a higher risk of death in patients with PPSC. PPSC, primary peritoneal serous carcinoma; ASOC, advanced serous ovarian cancer; HR, hazard ratio; CI, confidence interval

In addition, we performed subgroup analyses and illustrated the results with a forest plot (Fig. 4). When we compared the PPSC group with the matched ASOC group, patients who were between 50 and 69 years of age (HR 1.29, 95% CI 1.08–1.54, *P* = 0.005), were white (HR 1.18, 95% CI 1.03–1.35, *P* = 0.016), were married (HR 1.26, 95% CI 1.06–1.50, *P* = 0.008), had a tumor grade of III (HR 1.25, 95% CI 1.04–1.50, *P =* 0.016), had a tumor grade of IV (HR 1. 30, 95% CI 1.01–1.50, *P* = 0.041), were AJCC stage III (HR 1.20, 95% CI 1.02–1.41, *P* = 0.026), received chemotherapy (HR 1.23, 95% CI 1.08–1.41, *P* = 0.003) or underwent surgery (HR 1.26, 95% CI 1.10–1.44, *P* = 0.001) had more favorable outcomes in the matched ASOC group. Interestingly, patients with an unknown surgery status and those who did not undergo surgery had more favorable outcomes in the PPSC group (HR 0.68, 95% CI 0.47–0.99, *P* = 0.043). No significant differences were found between the other subgroups.

### Metastasis analysis

**Fig. 5:**
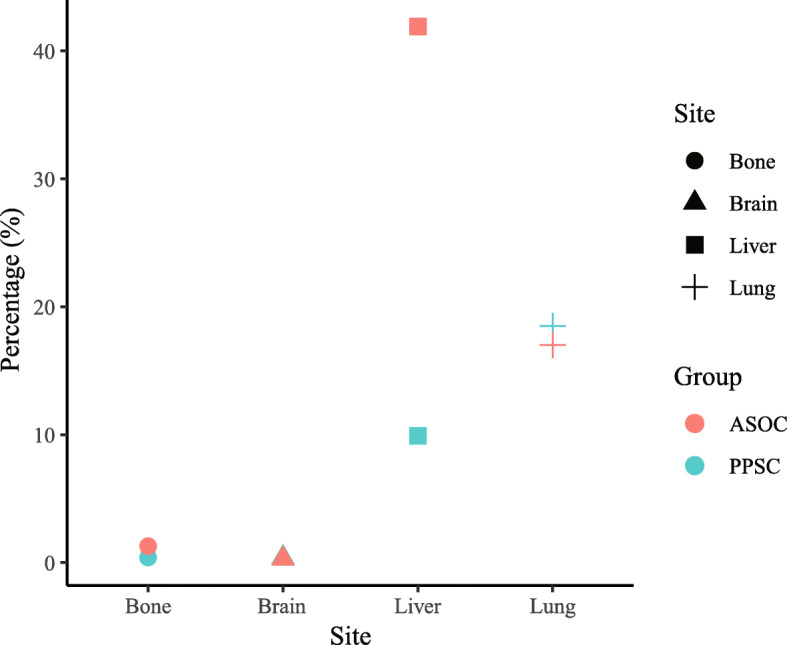
Site-specific metastasis distribution in PPSC and ASOC. PPSC, primary peritoneal serous carcinoma; ASOC, advanced serous ovarian cancer

In the PPSC group, 242 of 708 patients had distant metastasis at diagnosis, whereas in the ASOC group, 2376 of 7610 patients had distant metastasis at diagnosis. The distribution of the site-specific metastasis rate is presented in Fig. 5. In the PPSC group, the rates of site-specific metastasis were 0.4, 0.4, 9.9, and 18.5% for bone, brain, liver, and lung, respectively. In the ASOC group, the rates were 1.3, 0.3, 41.9, and 17% for bone, brain, liver, and lung, respectively.

## Discussion

This was a population-based retrospective study that was performed to determine the differences between PPSC patients and ASOC patients. Compared with ASOC patients, PPSC patients were more likely to be older and have stage IV disease but were less likely to be black, have high-grade tumors and receive surgical treatment. A previous study demonstrated that the survival outcomes of SOC patients were better than those of PPSC patients because SOC patients presented at a younger age and were more likely to have low-grade tumors and an early AJCC stage [18–20]. In this study, poorer OS was observed in PPSC patients than in ASOC patients. Even after the clinical features were balanced, the survival of PPSC patients remained inferior. The median OS of PPSC patients in this study was 36.0 months (95% CI 33.3–38.7), while it was 23.5 months (95% CI 18.6–39.8) in the report by Gamal [18] and 41.0 months (95% CI 30.0–55.0) in the report by Komiyama [2].

To further identify factors that affected OS in PPSC and ASOC, a matched subgroup analysis was performed. The PPSC group with HGSC, including grade III and grade IV disease, had an increased HR for death compared with that of the ASOC group. However, no difference in HR for death was found in patients with LGSC, including grade I and grade II. However, in the report by Gershenson, individuals with low-grade PPSC had a lower risk of death than individuals with low-grade SOC (HR 0.59, 95% CI 0.36–0.98; *P* = 0.04) [13]. Previous studies demonstrated that the origin and molecular features of HGSCs and LGSCs are completely different. HGSC may predominantly originate from serous tubal intraepithelial carcinoma and then implant into the ovary or peritoneum [21], while LGSC may originate from the epithelial cells that migrate from the fallopian tube to the ovary, after which they form a serous inclusion cyst and then develop into a serous cystadenoma [9]. Compared with that of HGSC, the p53 mutation or p53 expression frequency of LGSC was much lower, while the expression of estrogen receptor and progesterone receptor was higher.

At baseline, no significant difference was found in the AJCC stage distribution between the PPSC group and the ASOC group. In the univariate and multivariate analyses, both in the PPSC group and the ASOC group, patients with AJCC stage IV had a higher risk of death than those with stage III, and in both the AJCC stage III and IV subgroups, the OS of patients with PPSC was worse than that of patients with ASOC. In a systematic review, the survival time of patients with PPSC was shorter than that of patients with SOC in most clinical studies, but most of the differences did not reach statistical significance [22]. In some of the clinical studies, the tumor stages were not matched, and the sample sizes were limited. A study enrolled matched patients with ASOC and PPSC, and the results showed that the OS of the PPSC group was shorter than that of the ASOC group [23]. Older age and a higher proportion of patients with high tumor grade in the PPSC group might contribute to the worse outcome [20, 22].

We found that most subgroups favored ASOC patients except patient with no/unknown surgery treatment. In addition, the OS of PPSC patients remained worse after matching. All these proved that PPSC is a more aggressive cancer type compared to ASOC.

PPSC and SOC primarily spread within the abdominal cavity and have a low frequency of distant metastasis, especially in PPSC [24]. Most studies that focused on distant metastases of PPSC were case reports. One study reported a PPSC patient who had only inguinal lymph node metastasis [25]. Another study reported a PPSC patient with lung metastasis [3]. The most common site of distant metastasis in ovarian cancer is the liver, while lung, brain, and bone metastases are rare [26, 27]. In this population-based study, the total distant metastasis rates in the PPSC group and ASOC group were 34.2% (242/708) and 31.2% (2376/7610), respectively. A high frequency of liver and lung metastases were found in both the PPSC and ASOC groups, while bone metastasis and brain metastasis were rare.

Due to the low incidence of PPSC, the data are not sufficient to formulate a standard treatment plan. At present, the treatment of PPSC is consistent with that of SOC^11^. For stage III-IV ovarian cancer and primary peritoneal cancer, tumor cytoreductive surgery is recommended to decrease the maximum diameter of the residual tumor to less than 1 cm, and intraperitoneal/intravenous chemotherapy is recommended after surgery [28]. In addition, neoadjuvant treatment can be considered for patients with a high risk of surgery. A study reported that PPC patients had 21.2% (11/52 cases) of BRCA mutations [29]. For targeted therapy, those with BRCA1/2 mutations can be treated with bevacizumab or olaparib [30, 31]. Immunotherapy has provided promise for the treatment of advanced cancer, including ovarian cancer, but the role of immunotherapy in the treatment of PPSC remains unknown [32, 33].

This study has several limitations. First, some important clinical features, such as serum CA-125, weight, and residual tumor size after surgery, are not included in the SEER dataset. Second, the treatment regimen details are not specifically presented in the dataset. Next, no mutations information was presented in the SEER database. This may have a certain impact on the survival results since PARP inhibitors were effective in patients with BRCA mutations. Last, it’s hard to explain why patients with no/unknown surgery treatment had more favorable outcomes in the PPSC group. Some unknown reasons or confounding factors might lead to the result.

## Conclusion

This study demonstrated that the clinical features of PPSC patients and ASOC patients were clearly different. PPSC patients had an inferior OS compared with that of ASOC patients. In addition, most variables in the subgroup analysis were demonstrated to be adverse factors for PPSC compared with ASOC; hence, we propose that PPSC is more aggressive and has a poorer prognosis than ASOC.

## Supplementary Information

**Additional file 1.**

**Additional file 2.**

## Data Availability

The datasets used and/or analyzed during the current study are available from the publicly available SEER database.
