# Supplementary material for: Differences between primary peritoneal serous carcinoma and advanced serous ovarian carcinoma: a study based on the SEER database
Source: J Ovarian Res. 2021 Feb 27;14:40. doi: 10.1186/s13048-021-00788-y (PMC7916278; doi:10.1186/s13048-021-00788-y)
Supplement: Supplementary file 1 — Additional file 1. [file 13048_2021_788_MOESM1_ESM.docx]

Table 4, Clinical features of matched groups.

| Clinical features | PPSC N=708(%) | Match ASOC N=1416(%) | | Total N=2124(%) | P Value |
| --- | --- | --- | --- | --- | --- |
| Age |  |  |  | | 0.998 |
| ≤49 | 41 (6) | 81 (6) | 122 (6) | |  |
| 50-69 | 387 (55) | 775 (55) | 1162 (55) | |  |
| ≥70 | 280 (40) | 560 (40) | 840 (40) | |  |
| Race |  |  |  | | 0.864 |
| White | 600 (85) | 1201 (85) | 1801 (85) | |  |
| Black | 31 (4) | 68 (5) | 99 (5) | |  |
| Others | 77 (11) | 147 (10) | 224 (11) | |  |
| Marital status |  |  |  | | 0.842 |
| Married | 398 (56) | 800 (56) | 1198 (56) | |  |
| Not married | 283 (40) | 569 (40) | 852 (40) | |  |
| Unknown | 27 (4) | 47 (3) | 74 (3) | |  |
| Tumor grade |  |  |  | | 0.925 |
| Ⅰ | 18 (3) | 36 (3) | 54 (3) | |  |
| Ⅱ | 88 (12) | 163 (12) | 251 (12) | |  |
| Ⅲ | 318 (45) | 652 (46) | 970 (46) | |  |
| Ⅳ | 284 (40) | 565 (40) | 849 (40) | |  |
| AJCC stage |  |  |  | | 0.747 |
| Ⅲ | 466 (66) | 922 (65) | 1388 (65) | |  |
| Ⅳ | 242 (34) | 494 (35) | 736 (35) | |  |
| Chemotherapy |  |  |  | | 0.885 |
| Yes | 626 (88) | 1255 (89) | 1881 (89) | |  |
| No/Unknown | 82 (12) | 161 (11) | 243 (11) | |  |
| Surgery |  |  |  | | 0.492 |
| Yes | 649 (92) | 1310 (93) | 1959 (92) | |  |
| No/Unknown | 59 (8) | 106 (7) | 165 (8) | |  |

PPSC, primary peritoneal serous carcinoma; ASOC, advanced serous ovarian cancer; AJCC, American Joint Committee on Cancer.
